# Stability and degradation of (oxy)nitride photocatalysts for solar water splitting

**DOI:** 10.1039/d4su00096j

**Published:** 2024-05-02

**Authors:** Valérie Werner, Franky Bedoya Lora, Ziwei Chai, Julian Hörndl, Jakob Praxmair, Sandra Luber, Sophia Haussener, Simone Pokrant

**Affiliations:** a Department of Chemistry and Physics of Materials, Paris Lodron University Salzburg Jakob-Haringer-Str. 2A 5020 Salzburg Austria simone.pokrant@plus.ac.at; b Laboratory of Renewable Energy Science and Engineering, Ecole Polytechnique Fédérale de Lausanne 1015 Lausanne Switzerland; c Department of Chemistry, University of Zurich Winterthurerstrasse 190 CH-8057 Zurich Switzerland

## Abstract

Advancing towards alternative technologies for the sustainable production of hydrogen is a necessity for the successful integration of this potentially green fuel in the future. Photocatalytic and photoelectrochemical water splitting are promising concepts in this context. Over the past decades, researchers have successfully explored several materials classes, such as oxides, nitrides, and oxynitrides, in their quest for suitable photocatalysts with a focus on reaching higher efficiencies. However, to pave the way towards practicability, understanding degradation processes and reaching stability is essential, a domain where research has been scarcer. This perspective aims at providing an overview on recent progress concerning stability and degradation with a focus on (oxy)nitride photocatalysts and at providing insights into the opportunities and challenges coming along with the investigation of degradation processes and the attempts to improve the stability of photocatalysts.

## Abstract 2

The investigation of degradation processes provides opportunities for the stability improvement of photocatalysts and photoelectrodes. This perspective provides an overview on recent progress with a focus on (oxy)nitrides.

## Graphical abstract

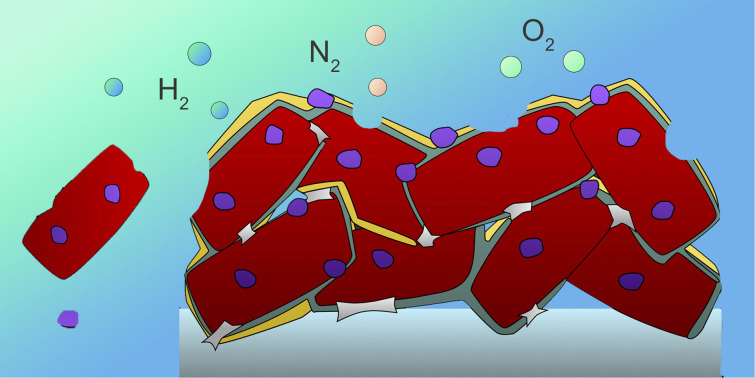

**Sustainability spotlight**

The potential of hydrogen as a green energy carrier remains latent, since 96% of the world's hydrogen production (95 Mt in 2023) is still derived from fossil-fuels. Photocatalytic and photoelectrochemical systems not only allow sustainable hydrogen production but also give the opportunity to efficiently harvest, convert and store abundant solar energy. One of the major challenges hindering these conversion systems from becoming economically feasible is the insufficient long-term stability. This perspective addresses the importance to tackle this challenge to inspire future research work and fuel co-operations. It pays particular attention to a visible light absorbing materials class, the (oxy)nitrides, that holds promise because of high efficiencies, but poses special challenges with respect to degradation because of its mixed-anion character.

## 1 Introduction

Coping with the increasing energy demand whilst tackling environmental challenges requires a transition from fossil energy carriers to green energy carriers and an expanded exploitation of low- and zero-carbon energy sources.^1,2^ Hydrogen is stated to be a promising green energy carrier of the future since it can be readily converted into electricity *via* fuel cell technology or be used as a feedstock in the chemical industry.^3,4^ However, to date hydrogen production processes (95 Mt in 2023 ^5^), primarily based on fossil fuels, have been associated with a significant emission profile.^6,7^ Therefore, numerous efforts have been made over the past decades to advance alternatives aiming for sustainable hydrogen production.^1,8–11^ Utilizing the photocatalytic properties of some semiconducting materials to cleave water into hydrogen and oxygen has proven to be a promising approach in this context and has led to the development of photocatalytic and photoelectrochemical (PEC) systems.^8,12^ The latter one refers to a system where the photocatalytically active material is immobilized on a substrate forming a photoelectrode, that is electrically connected to another (photo)electrode within a closed circuit, whilst in photocatalytic systems the photocatalyst is suspended in an aqueous medium.^8^ In order to be economically competitive with the conventional production routes of hydrogen, early techno-economic studies predict that a system efficiency of 20% over a lifetime of 10 years is required for PEC systems and a system efficiency of 10% over 5 years is required for photocatalytic systems.^13^ A more recent study suggests that solar-to-hydrogen (STH) efficiencies must be higher than 27% with lifetimes of at least 2 years for PEC cells to be economically viable, while a STH efficiency higher than 6% is needed for a photocatalytic systems.^14^ Additionally, the cost target for hydrogen has been recently confirmed to be *ca.* $2 per H_2_ kg.^15^

As most of the research has been devoted to the development of photocatalytic materials and device setups to reach higher efficiencies, the challenges that arise from degradation during long-term stability testing are still insufficiently explored for most of the available material systems.^11^ In terms of stability, oxide photocatalysts, *e.g.* TiO_2_ or SrTiO_3_, are considered to show a high resistance toward photocorrosion.^16–18^ For instance, this has been demonstrated by Lyu *et al.* for Al-doped SrTiO_3_ maintaining stable overall water splitting for more than 1000 h under constant illumination.^19^ However, the efficiency of this system remained with a solar-to-hydrogen efficiency of 0.3% far off of the requirements for the practical application of photocatalytic systems.

Moreover, most of the metal oxide photocatalysts exhibit wide band gaps limiting light absorption to the ultraviolet range which corresponds to only 4% of the solar spectrum.^20,21^ Visible light absorbing, narrow band gap (<3 eV) photocatalysts allow more efficient light harvesting which positively affects the system efficiency.^8,22,23^ Typical material classes are narrow band gap oxides (*e.g.* WO_3_, α-Fe_2_O_3_, BiVO_4_) as well as non-oxide or mixed-anion materials (nitrides, oxynitrides, sulfides *etc.*) For example (oxy)nitrides exhibit a narrower band gap compared to their related metal oxides due to the contribution of N 2p orbitals to the valence band maximum (VBM).^24–26^ Even though narrow band gap materials have demonstrated great potential to reach higher efficiencies, the stability challenge remains.^27,28^ The lifetime of these photocatalysts in photocatalytic and PEC systems is usually in the range of minutes or several days, an insufficient stability for a practical application.^16,17,27–32^

So far, few studies have concentrated on investigating degradation mechanisms, such as Toma *et al.* on BiVO_4_,^33^ to provide a rational basis for stability improvement. Nevertheless, measures to improve the stability of photocatalysts, *e.g.* by applying cocatalysts or tuning the preparation method, are constantly developed and led to stable performances for up to 1000 h in case of BiVO_4_ or hematite (α-Fe_2_O_3_), respectively.^34,35^ Also in case of non-oxide and mixed-anion photocatalysts which show an increased risk of photocorrosion due to self-oxidation, attempts have been conducted to address this challenge.^22,27,36,37^ For instance, in case of TaON photoanodes the stability was improved by preventing self-oxidation through CoO_*x*_ cocatalyst loading onto TaON particles prior to electrode preparation.^36^ However, the applied testing duration did not exceed a few hours. A procedure for measurement of long-term stability based on chronoamperometric measurements in PEC systems was only recently proposed by Vanka *et al.* based on their studies on Pt-decorated GaN nanostructures on an n^+^–p Si photocathode^38,39^ and extended by Zhang *et al.*, who suggested to rely on *in operando* dissolution measurements for thicker photoabsorbers.^40^

**Fig. 1:**
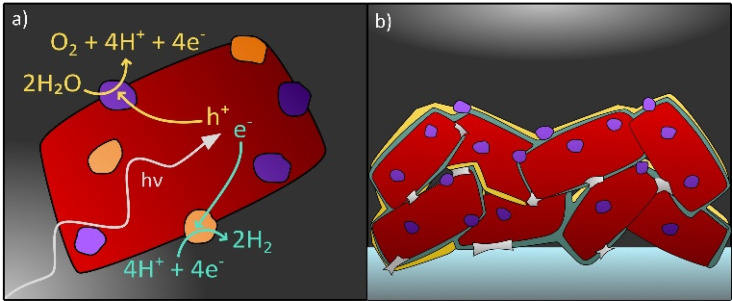
Photocatalyst particle(s) (a) suspended in acidic aqueous electrolyte and (b) deposited on a conducting substrate (dark grey) forming a PEC electrode. Cocatalyst particles are coloured in orange and violet, protective layers in yellow and necking in light grey.

Even though over the past decade the call for standards regarding stability testing has increased and research about degradation of photocatalysts has intensified,^38^ the topic of degradation in photocatalytic and PEC systems still remains a considerable challenge to overcome. An in-depth understanding of degradation processes is necessary to find solutions to prolong the lifetime of photocatalysts in water splitting applications and beyond. This perspective provides an overview on the recent progress in the field of stability of photocatalysts for solar water splitting with the primary focus on (oxy)nitrides. In this context, known material degradation processes and factors that influence the stability of photocatalyst particles in both application forms, *i.e.* suspended in water or integrated in a PEC device shall be reviewed (see Fig. 1). Possible strategies to investigate degradation by *in situ* and *ex situ* techniques are reviewed. Then, system specific degradation as a function of the device set-up and the operating conditions is discussed with a focus on PEC water splitting. In addition, possible pathways to improve the stability of photocatalysts are outlined.

## 2 Material degradation

The functionality of photocatalytic and PEC systems relies on the photocatalytic activity of a semiconductor towards either one or both of the water splitting half reactions, the oxygen evolution reaction (oxidation, OER) and the hydrogen evolution reaction (reduction, HER).^8,16,41^ For a semiconductor to be used as a photocatalyst for solar water splitting, the material needs to exhibit suitable optical properties for efficient light harvesting, facile charge carrier separation and transport, as well as suitable band edge positions with regard to either one or both of the water splitting half reactions.^22,42,43^ The latter one refers to the thermodynamic requirement, that the potential associated with the conduction band minimum (CBM) should be more negative with respect to NHE than the water reduction potential and/or the VBM potential should be more positive with respect to NHE than the water oxidation potential.^16,44^

When irradiated with light, photogenerated charge carriers can drive the corresponding water splitting half reaction, HER (electrons) and OER (holes).^44–46^ However, in addition, undesired processes can take place that lead eventually to performance decrease. They are summarized by the term “degradation”.

In general, the degradation of photocatalysts and/or photoelectrodes in photocatalytic and PEC devices can primarily originate from:

(i) Charge-related degradation: corresponds to material corrosion including a charge transfer (*e.g.* photocorrosion: photogenerated minority charge carriers are involved in the corrosion reaction).

(ii) Chemical degradation/corrosion: deterioration of the photocatalyst due to the local electrolyte environment at the semiconductor–electrolyte interface *e.g.* dissolution or poisoning.

(iii) Mechanical degradation of photocatalysts and photoelectrodes including loss of cocatalysts or photocatalyst particles.

Especially in photon driven devices, photogenerated charge carriers can participate in corrosion processes resulting in the degradation of the photocatalyst.^47,48^ This degradation *via* photocorrosion describes the process where the photogenerated charge carriers participate in the self-oxidation or self-reduction of the semiconductor instead of driving the water splitting half reactions and is often accompanied by a performance drop with increasing irradiation time.^29,46,48,49^ In PEC devices a thermodynamic stability criterium can be formulated: if the HER potential is more positive *versus* NHE than the cathodic decomposition potential of the photocatalyst or if the OER potential is more negative *versus* NHE than the anodic decomposition potential at the given operational conditions, the photocatalyst is thermodynamically stable. Otherwise, and if kinetics of the HER or OER is unfavourable compared to the decomposition reactions, photocorrosion proceeds competitively with the water splitting half reactions.

The competition of photocorrosion and the water splitting reaction in PEC devices has been thoroughly discussed in previous reports of Nandjou and Haussener.^49–51^

Oxides like BiVO_4_ are known to degrade by oxygen loss (eqn (1)) instead of driving the OER.^33^

1 2O^2−^ + 4h^+^ → O_2_

In mixed-anion systems, specifically in oxynitrides, the presence of nitrogen anions in the structure causes that an additional path to self-oxidation is available. In the process of self-oxidation (eqn (2)), photogenerated holes oxidize nitrogen anions to molecular nitrogen.^52^

2 2N^3−^ + 6h^+^ → N_2_

Indeed, (perovskite-related) oxynitrides have been found to release nitrogen gas in the early stages of catalytic reactions.^24,53^ In addition, it has been observed experimentally that the self-oxidation in (oxy)nitrides proceeds competitively with the OER and causes a deactivation of the photocatalyst, resulting in a (rapid) performance drop.^36,52,54–57^ For instance, this has been found in case of LaTiO_2_N photoanodes and is reflected by a rapid decrease in photocurrent within a few minutes and a decrease in the O_2_ evolution over time.^56^ However, the photocorrosion of oxynitrides does not necessarily lead to immediate performance decrease from the beginning.^58^ Density functional theory (DFT) computational studies on SrTaO_2_N and SrNbO_2_N photocatalysts indicate that under OER catalytic conditions, nitrogen atom vacancies left on the surface due to the decomposition of the material into nitrogen gas are often healed by oxygen atoms.^58,59^ For SrTaO_2_N, the highest overpotential of the four-step OER reaction decreases proportional to the replacement of surface nitrogen atoms by oxygen atoms.^58^ However, for SrNbO_2_N, as the amount of nitrogen atom replacement increases, the OER activity initially increases and then decreases.^59^

Besides charge-related degradation, the material can also undergo chemical degradation due to corrosion processes taking place at the interface to the electrolyte without any light irradiation or electrical bias.^60,61^ The long-term exposure to the aqueous environment causes substantial challenges to the photocatalyst. An indicator for the stability of a material against chemical corrosion at a certain pH and potential range can be obtained from Pourbaix diagrams.^62^ Studies on α-Fe_2_O_3_ showed that it becomes chemically unstable in electrolytes at low pH whereas it exhibits stability in (near-)neutral and alkaline electrolytes.^29,63–65^ (Oxy)nitrides, however, show an immaculate chemical stability even in strongly alkaline environment (pH ≥ 13).^26,66–68^ Moreover, chemical corrosion can also be accelerated by illumination (photochemical corrosion) driving for example the dissolution of BiVO_4_ even though stability towards photocorrosion under water splitting condition is predicted.^33,46^ Several studies discussed the dissolution process of oxide photocatalysts such as ZnO, Cu_2_O, BiVO_4_, WO_3_ and α-Fe_2_O_3_, but it has not yet been investigated in detail for (oxy)nitrides.^29,69–72^

In the context of PEC and photocatalysis, mechanical degradation has been discussed mostly with respect to PEC photoelectrodes, *i.e.* the degradation due to mechanical erosion.^35^ Moreover, for photoelectrode preparation, commonly a thin layer in a size range from a few nanometres to several micrometres is deposited on a conductive substrate.^12,35,73,74^ The preparation method hereby influences strongly the mechanical stability of the thin layer which can be for instance a compact thin film or can compose of individual particles that are arranged randomly.^75,76^ An intermediate format are precursor films that form nanostructures during their transformation to the final composition such as nanorods in the case of Ta_3_N_5_.^77–80^ Although compact films could start to peel off during long-term measurements which presumably causes a significant drop in performance due to the decreased contact between photoactive material and substrate, thin film technologies result in photoelectrodes with a higher mechanical stability than particle-based films.^12^ However, by performing a necking step where *e.g.* TiO_2_ necks are formed between the particles, the mechanical interparticle contact as well as the contact between the particles and the substrate are improved.^73,81,82^ Evidently, based on these differences, the mechanical degradation of thin films and particle-based photoelectrodes is expected to proceed divergently.

Concluding, degradation results in changes in the composition and morphology of the photocatalyst particles and photoelectrodes at several length scales. Suitable characterization methods are required to investigate degradation processes of photocatalysts for solar water splitting and will be discussed in the following section.

## 3 Methods and standards to investigate stability and degradation and their application to oxynitrides

In order to assess the stability of a photocatalyst in photocatalytic or PEC water splitting devices, a consensus about the measurement and testing conditions needs to be found in order to define a guideline. Within the last few years, several perspectives and reviews have been published proposing standards and guidelines for assessing the short-term and long-term stability of photocatalysts and photoelectrodes.^38,48,83,84^

**Fig. 2:**
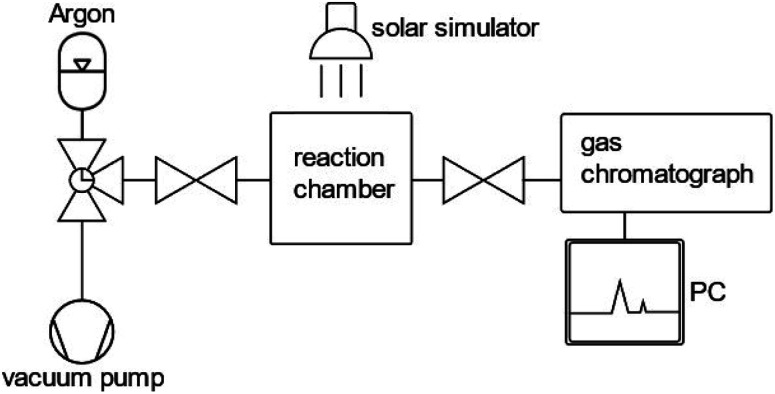
Schematic representation of an experimental set-up suitable for photocatalytic performance assessment. The gas chromatograph allows the determination of oxygen and hydrogen concentrations in the reaction chamber.

The most significant performance indicator for photocatalytic systems is the STH efficiency.^85^ However, the accessibility of this quantity is limited to systems that are able to perform the full water splitting reaction. When only one half reaction (OER or HER) is examined, the photocatalytic activity is commonly investigated by means of either oxygen or hydrogen gas evolution rate or by photocurrent density at a given bias voltage in photocatalytic and PEC measurements, respectively.^20,44,86^ With regard to photocatalytic systems, the gas evolution rates are commonly determined by measuring the oxygen or hydrogen concentration in a closed vessel filled with inert gas, such as argon, over a time span of two to five hours for (oxy)nitride photocatalysts. A schematic representation of an experimental set-up to determine hydrogen and oxygen evolution rates is displayed in Fig. 2.

When the performance with respect to half reactions is evaluated, sacrificial agents are necessary, that act as electron (for example Ag^+^ for the OER) or hole (for example methanol for the HER) scavengers, respectively.^87^ For stability testing the gas evolution can be detected over a long-term (*e.g.* over 24 h) photocatalytic measurement.^88,89^ However, a more frequently used approach are cycling tests, which is a repetition of short-term measurement intervals (*e.g.* 3 h) with intermittent product removal (evacuation) and electrolyte exchange.^90–92^ After a measurement, the photocatalyst is removed from the electrolyte and redispersed in a fresh electrolyte while the reaction chamber is completely evacuated before the next measurement is started. This approach allows the observation of long-term stability of a photocatalyst without taking into account effects caused by the accumulation of products (*e.g.* favouring back reactions) and the electrolyte composition (*e.g.* presence of sacrificial agent).^93^ Considering oxygen evolution rates, it must be stated that the performance of oxynitrides does not depend exclusively on the nominal bulk composition, but also on the exact stoichiometry, crystallinity, the exposed facets/surfaces, the cocatalyst systems and the electrolyte. In the case of LaTiO_2_N oxygen evolution rates between 25 μmol h^−1^ and 260 μmol h^−1^ as a function of particle morphology and cocatalyst decoration have been reported.^94^ Concerning long term stability a slight drop in hydrogen evolution rate for Pt decorated LaTiO_2_N catalysts in the course of four 25 min measurement cycles was reported^52^ and no drop in oxygen and hydrogen evolution rate for RhCrO*_y_* and CoOOH decorated LaTiO_2_N in the course of four 5 h measurements.^95^ Hence, one of the difficulties to assess consistently and comparably O_2_ or H_2_ evolution rates, and therefore indirectly stability, is that even when fixing the material composition there is still a large parameter space which needs to be controlled and reported to assure reproducibility. Some performance indicators of oxynitride photocatalysts have been summarized in Table 1.

**Table 1:** Oxygen and hydrogen evolution rates of selected oxynitride and nitride photocatalysts

Photocatalyst system	Amount of catalyst (g)	Electrolyte	Light source	O_2_ evolution rate (μmol h^−1^)	H_2_ evolution rate (μmol h^−1^)	Ref.
CoO_*x*_/LaTiO_2_N	0.1	40 mM AgNO_3_ + La_2_O_3_ buffer	300 W Xe lamp, *λ* > 420	260	—	94
LaTiO_2_N	0.2	10 mM AgNO_3_ + La_2_O_3_ buffer	300 W Xe lamp, *λ* > 420	10.2	—	52
IrO_2_/Ca_0.25_La_0.75_Ti_2.25_N_0.75_	0.2	10 mM AgNO_3_ + La_2_O_3_ buffer	300 W Xe lamp, *λ* > 420	500	—	52
CoO_*x*_/LaTiO_2_N	0.3	0.05 M AgNO_3_ + La_2_O_3_ buffer	300 W Xe lamp, *λ* > 420	681	—	95
RhCrO_*y*_/CoOOH/LaTiO_2_N	0.3	H_2_O	300 W Xe lamp, AM1.5G	2.4	4.7	95
CoO_*x*_/LaTiO_2_N	0.2	0.05 M AgNO_3_ + La_2_O_3_ buffer	300 W Xe lamp, *λ* > 420	736	—	68
CoO_*x*_/BaTaO_2_N	0.1	0.01 M AgNO_3_ + La_2_O_3_ buffer	300 W Xe lamp, *λ* > 420	134.6	—	96
TiO_2_/SiO_2_/RhCrO_*x*_/LaMg_1/3_Ta_2/3_O_2_N	0.2	H_2_O	300 W Xe lamp, *λ* > 300	11	22	97
CoO_*x*_/Ta_3_N_5_	0.1	0.05 M AgNO_3_ + La_2_O_3_ buffer	300 W Xe lamp, *λ* > 420	287.3	—	98

For PEC systems, the stability is determined by measuring the dissolution products, as proposed very recently,^64^ or, more frequently, by recording the decrease in photocurrent over time. For this purpose, chronoamperometry under illumination is performed to track the photocurrent as a function of time.^99^ In addition, the evolved gases should be quantified in order to calculate the Faraday efficiency of the system.^100^ When the Faraday efficiency is close to 100%, it indicates that the photogenerated charge carriers are fully utilized to drive the water splitting reaction.^8^ For (oxy)nitrides, the Faraday efficiency is usually higher than 90%.^32,101–104^ For instance, a Faraday efficiency close to unity (99.2%) regarding the OER was demonstrated for BaTaO_2_N decorated with CoO microflowers and of 98% for Ta_3_N_5_.^102,105^ However, faradaic efficiency values ranging between 76% and 90% have also been reported, which indicates that side reactions (back reaction, corrosion) that compete with the water splitting reactions take place.^81,82,106^ Moreover, the measurement duration is mainly limited to a couple of hours in case of (oxy)nitrides, which is assumed to be too short to give a statement about the long-term stability of a system.^32,81^

Investigating oxynitrides special attention must be paid to nitrogen when tracking the gas evolution during photocatalytic and PEC measurements. Indeed nitrogen evolution has been reported multiple times during performance and stability assessments of (oxy)nitrides.^52,74,82,96–98^ For example for LaTiO_2_N Kasahara *et al.* estimated that 12% of the generated holes went into N_2_ evolution (for 440 μmol O_2_, 40 μmol N_2_),^52^ while for various morphologies with and without cocatalyst decoration achieving a broad range of O_2_ evolution rates (15–736 μmol h^−1^), N_2_ evolution in the order of 2–8 μmol was reported during the first hours.^68^ Also Wang *et al.* observed substantial N_2_ evolution during the first two of four 5 h photocatalytic full water splitting cycles performed by LaTiO_2_N.^95^ This observation is attributed to the corrosion *via* photo-oxidation of the material which is correlated to the loss of nitrogen from the structure, that seems to stop after one or two hours. Similar observations were reported for BaTaO_2_N.^96^ During chronoamperometric PEC measurements of LaTiO_2_N containing electrodes, small quantities of N_2_ evolution were detected as well.^101,106^ Qualitatively the reports concerning small quantities of N_2_ evolution from oxynitrides during the first one or two hours of operation are consistent. However, since quantitative measurements are scarce and systematic studies are missing, it is difficult to assess which parameters apart from the nominal bulk composition are important for N_2_ evolution. Since the determination of the full parameter space that is responsible for degradation is still under investigation, it is not surprising that quantitative and even sometimes qualitative degradation behaviour of the same materials is measured differently by different groups.

Since stability testing just based on short- and long-term performance measurements is insufficient to understand degradation processes, *ex situ* characterization is required to thoroughly investigate structure, composition, and morphology before and after stability testing (post-mortem). Even more information is obtained by *in situ* characterization enabling the observation of material property evolution during the testing phase. In practice, *in situ* characterization is more complex and is not readily available for all systems.

A large variety of characterization methods has been successfully applied to study degradation processes including bulk, surface and liquid phase analytics. Concerning bulk characterization techniques, the assessment of the crystal structure by X-ray diffraction (XRD) prior stability testing and post-mortem has been carried out. Complementary information is obtained by microscopic techniques such as secondary and transmission electron microscopy (SEM and TEM) as well as atomic force microscopy (AFM). These techniques are vital to reveal changes in morphology of the photocatalyst or photoelectrode.^33,39,40,107,108^ Compositional changes can be identified by spectroscopic methods such as X-ray photoelectron spectroscopy (XPS) and TEM/SEM energy dispersive X-ray spectroscopy.^37,109–112^ For example nitrogen loss was confirmed by a reduced nitrogen signal detected *via* XPS after PEC measurements performed on LaTiO_2_N electrodes.^56^ Moreover, with respect to *ex situ* and more importantly *in situ* characterization, thin film photoelectrodes are advantageous due to their well-defined and atomically flat surfaces allowing the use of dedicated surface characterization technques.^74^ For instance, *ex situ* and *in situ* neutron reflectometry and grazing-incidence X-ray absorption spectroscopy are used to explore surface modifications of oxynitride thin films.^109,113^ The study on SrTaO_*x*_N_*y*_ showed, that the oxynitride surface undergoes a compositional change due to the dissolution of SrO_*x*_ into the electrolyte enriching Ta and lattice Sr at the surface and the slight loss of nitrogen from the structure.^109^ With respect to LaTiO_*x*_N_*y*_ the slight loss of nitrogen is predominantly found within the first 3 nm from the surface.^113^

Moreover, the investigation of the electrolyte can contribute to identifying the corrosion of system components (*e.g.* cell housing) as well as dissolution of the photocatalyst itself. One of the most prominent liquid phase characterization techniques in this context is the inductively coupled plasma mass spectrometry (ICP-MS) (*ex situ* and *in situ*). The Cherevko group developed a setup combining ICP-MS with an illuminated scanning flow cell which allows the monitoring of dissolution processes and changes of the electrolyte composition whilst performing PEC measurements.^60,61^ With this technique the photocorrosion processes of BiVO_4_,^40^ WO_3_ (ref. 70) and hematite^29^ have been investigated in various electrolytes.

**Fig. 3:**
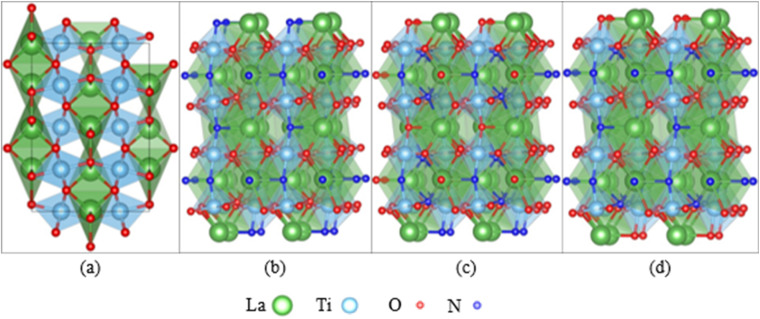
(a) LaTiO_2_N in the orthorhombic cell observed experimentally, featuring disorder in the positions of oxygen and nitrogen. LaTiO_2_N (001) surface models with (b) linear *trans*-chain ordering, (c) 2D *cis*-chain ordering, and (d) mixed *trans*- and *cis*- ordering of the Ti–N–Ti bonds.

Besides the experimental approach to assess the degradation of photocatalysts, theoretical studies are essential to complement and understand the findings of stability testing. The degradation of oxynitride surfaces in contact with electrolytes can be understood at the atomic scale using computational methods, such as the study on corrosion-induced surface alterations (*e.g.*, dissolution, reconstruction).^58,59^ Existing research on the interfaces between oxynitride and electrolytes has focused on their catalytic properties, although the degradation of oxynitride surfaces is deeply intertwined with their interface geometry and electronic structure. Studying the interfaces between oxynitride and electrolytes using computational methods is more complex compared to studying the interfaces between oxides and electrolytes. Firstly, for a fixed chemical composition, an exposed surface of oxynitrides can exhibit various possible anion distributions with different stabilities, such as disordered, cis and trans configurations (see Fig. 3). In DFT simulations, identifying the most stable surface and surface structure of oxynitrides requires exploring a larger structural space compared to determining the most stable surface and surface structure of oxides.^114–116^ Some computational studies have shown that cations can influence the anion order.^117^ Different anion orders can lead to significant differences in properties related to catalytic activity, such as optical properties, hole effective mass, and overpotential.^117–121^ Additionally, oxynitrides with varying N and O ionic composition ratios or cationic components give rise to a wide variety of mechanical, electronic, and optical properties.^58,59,122–124^ High-throughput screening combined with DFT calculations can be used to explore the vast space of chemical compositions and structures, and to rapidly screen for potential oxynitrides with optimal properties for a given chemical composition.^116,125^

In summary, the stability of photocatalysts is assessed based on short- and long-term performance measurements. A thorough characterization of the structure/composition and morphology of the photocatalyst before and after the photocatalytic or PEC measurement is essential to identify changes due to degradation. Theoretical studies hold a great potential to explain macroscopically observed degradation on the atomistic level. Moreover, the application of *in situ* characterization techniques offers the possibility to follow degradation under operating conditions.

## 4 System specific degradation: electrode set up, operating conditions, performance and degradation of oxynitride PEC electrodes

Regarding system specific degradation in photocatalytic and PEC applications, it is important to consider that the local electrolyte composition near the solid–liquid interface constantly undergoes changes during operation (pH shift, gas evolution). Furthermore, this effect is a function of the microenvironment due to transport processes, even if the device geometry and the operating parameters are kept constant. In addition, the stability of the system can be affected differently when subjected to varying macroscopic operation conditions such as light intensity, photon-driven bias, electrical bias, temperature variations or exposure to corrosive environments. In both cases, the caused charge related degradation and chemical degradation might vary as a function of the employed system parameters and operating conditions. The same is true for mechanical degradation, such as the decomposition of the photoelectrode, that can be affected differently as a function of device operating conditions, *e.g.* electrolyte, flow, or the exact synthesis route used for their fabrication.

Oxynitride photoelectrodes are typically fabricated by two different methods: (i) particle deposition either by particle transfer^56^ or *via* electrophoresis^57^ and (ii) epitaxial thin film growth, *e.g. via* pulsed layer deposition.^126^ The particle-based approaches are the most commonly reported in the literature, usually outperforming thin films in terms of photocurrent densities. Due to the roughness of particle based films, the surface area being in contact with the electrolyte (solid–liquid interface) is significantly enhanced resulting in higher photocurrents.^74,76^ Although the syntheses of thin film oxynitrides, using pulsed reactive crossed-beam laser ablation and radio-frequency magnetron sputtering,^76,127,128^ are rather complex and the resulting photoelectrodes suffer from low photocurrents and limited scalability, epitaxially-grown films can be very useful to investigate the activity of the exposed crystal facets on the performance for water splitting. For example, Burns *et al.* found that (011) oriented LaTiO_2_N films exhibited 30% higher photocurrents than those with (001) orientation, mostly due to a faster extraction of charges derived from photons in the visible region.^129^ The dilemma “particles based *vs.* thin films” has been discussed to a great extent by Lippert *et al.*^74,76^ Briefly, particle-based systems have 10 to 20 times higher surface area and better photon absorption due to their micro-scale features compared to the nano-scale thicknesses of thin films. This results in particle-based photoelectrodes achieving photocurrents more than 10 times higher than their thin film counterparts. This behaviour has been observed for LaTiO_*x*_N_*y*_, BaTaO_*x*_N_*x*_, CaNbO_*x*_N_*y*_^76^ and SrTaO_2_N^130^ photoelectrodes. Hence, from a performance perspective, there is currently little incentive to use oxynitride thin films. In addition, so far there have been no reports of chronoamperometries, or similar studies, for epitaxially-grown oxynitride thin films, which could bring insights into the effect of crystal orientation on the degradation mechanism. It is proposed that charge transfer extraction is slower for the orientation (001),^129^ and this could translate into hole accumulation and faster degradation, although this has yet to be tested experimentally. Therefore, we will focus the discussion of degradation of oxynitrides on particle-based approaches such as electrophoretic deposition and the particle-transfer method for the preparation of particle-based photoelectrodes providing inexpensive and scalable alternatives to thin film depositions.^56,57,81,82,131–133^

An important factor for the set-up of particle-based electrodes with respect to performance and stability is particle morphology. Hojamberdiev *et al.* suggest that increasing the particle size of BaTaO_2_N, from 2 to 12 μm, leads to decreased photocurrent densities due to longer travel distances for the holes and electrons.^96^ However, when the particles were too small and surface area was increased, the higher presence of boundaries promoted surface recombination. Therefore, morphology could play a critical role on the degradation rates; fewer interparticle boundaries and micrometre-sized particles are preferred, while high surface area could increase the degradation rates due to increased nitrogen release from the surface. Unfortunately, Hojamberdiev *et al.* did not report chronoamperometries, making the comparison of degradation rates for different morphologies very difficult. Feng *et al.* and Landsmann *et al.* arrived at similar conclusions when comparing LaTiO_2_N particles produced *via* solid–state reactions and polymerized complex methods.^81,134^ The former method produced larger particles with better crystallinity, which resulted in higher photocurrent densities.

**Fig. 4:**
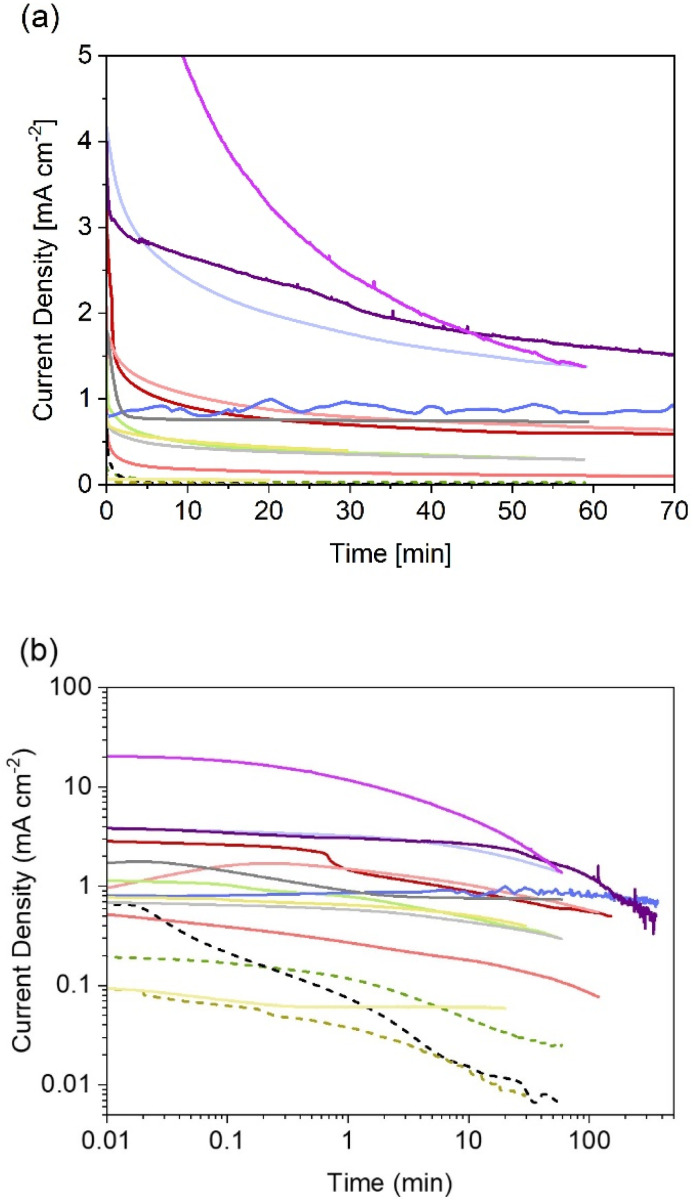
Reported (photo)current densities at constant electrode potential for different systems using particle-based oxynitride photoelectrodes. (a) Data with linear axes up to 70 min, and (b) in log-scale for easier comparison between all sets of data dashed lines indicate photoelectrodes without cocatalysts. More experimental conditions, sources and colour codes can be found in Table 2.

Thanks to the higher number of reports on particle-based photoelectrodes compared to thin films, more studies can be found on the stability of these systems. Notwithstanding the current research focus on improving photocurrents and faradaic efficiencies, some studies include chronoamperometries recorded during the first hour of photoelectrolysis (see Fig. 4). Table 2 show the chronoamperometries for different oxynitrides as reported in the literature for various oxynitrides and at various operating parameters, including two original measurements made by the authors for LaTiO_2_N performing under irradiation of 1 and 130 suns (1 sun = 1 kW m^−2^).

**Table 2:** Summary of reported chronoamperometries for particle-based oxynitride photoelectrodes. Retention is calculated as the percentage of photocurrent after 60 min with respect to the initial photocurrent and the photocurrent after 10 min^a^

Deposition	Photoelectrode	Electrolyte	*V*_RHE_	Light source	*j*_(*t*=0)_ (mA cm^−2^)	*j*_(*t*=10 min)_ (mA cm^−2^)	PR_0_@60 min (%)	PR_10 min_@60 min (%)	Colour code Fig. 4	Ref.
EPD	FTO|LaTiO_2_N|Co_3_O_4_	1 M NaOH pH 13.6	1.23	AM1.5G	3.3	0.90	18	66	d. Red	81
EPD	FTO|LaTiO_2_N|Co_3_O_4_	1 M NaOH pH 13.6	1.23	AM1.5G	1.3	0.18	8	60	Red	137
EPD	FTO|Na_0.1_La_0.9_TiO_2.2_N_0.8_|Co_3_O_4_	1 M NaOH pH 13.6	1.23	AM1.5G	2.1	1.0	31	63	l. Red	137
PT	FTO|SrTaO_2_N	1 M NaOH pH 13.6	1.23	AM1.5G	0.2	0.046	13	54	d. Green	130
PT	FTO|SrTaO_2_N|CoPi	1 M NaOH pH 13.6	1.23	AM1.5G	1.5	0.49	20	61	l. Green	130
PT	Ti|Ta|BaTaO_2_N|Co	0.2 M KPi pH 13	0.8	AM1.5G	0.93	0.85	91	101	d. Blue	135
PT	Ti|Ta|BaTaO_2_N|FeO_*x*_|Co(OH)_*x*_	0.5 M KBi pH 13	1.23	AM1.5G	4.2	2.4	33	58	l. Blue	138
EPD	Ti|TaON	0.1 M Na_2_SO_4_ pH 6	0.95	300 W Xe lamp	0.44	0.016	1	38	Black	36
EPD	Ti|TaON|CoO_*x*_	0.1 M Na_2_SO_4_ pH 6	0.95	300 W Xe lamp	1.5	0.76	49	97	d. Grey	36
EPD	Ti|TaON|IrO_*x*_	0.1 M Na_2_SO_4_ pH 6	0.95	300 W Xe lamp	0.73	0.44	41	69	l. Grey	36
EPD	Ti|TaON|CoO_*x*_	0.1 M Na_2_SO_4_ pH 8	1.07	300 W Xe lamp	1.8	0.76	41	97		36
EPD	Ti|TaON|CoO_*x*_	0.1 M PO_4_^−3^ buffer	1.07	300 W Xe lamp	2.1	2.0	100	110		36
EPD	FTO|LaTiO_2_N	0.1 M Na_2_SO_4_ pH 13.4	1.20	AM1.5G	0.12	0.014	N/A	N/A	Brown	134
EPD	FTO|LaTiO_2_N|CoO_*x*_	0.1 M Na_2_SO_4_ pH 13.4	1.20	AM1.5G	0.85	0.51	N/A	N/A	Yellow	134
EPD	FTO|LaTiO_2_N|CoO_*x*_	0.1 M Na_2_SO_4_ pH 13.4	1.20	0.034 suns	0.096	0.060	N/A	N/A	l. Yellow	134
EPD	FTO|LaTiO_2_N|Co_3_O_4_/NiO_*x*_	0.1 M Na_2_SO_4_ pH 13.4	1.23	AM1.5G	4	2.7	40	60	Violet	This work
EPD	FTO|LaTiO_2_N|Co_3_O_4_/NiO_*x*_	0.1 M Na_2_SO_4_ pH 13.4	1.23	∼130 suns	21	4.8	7	29	Pink	This work

a EPD: electrophoretic deposition, PT: particle transfer, PR_0_: photocurrent retention respect to initial photocurrent, PR_10min_: photocurrent retention respect to photocurrent after 10 min, d.: dark; l. light.

As it can be noted from Fig. 4, only one published study has reported the evolution of photocurrent densities during 6 hours.^135^ In that study, BaTaO_2_N particles, supported on Ta and covered with CoPi cocatalyst, exhibited relatively stable photocurrents with 20% decrease after 6 hours of continuous operation under 1 sun. The authors also recorded a faradaic efficiency near 100% for oxygen evolution, so it was unclear if the degradation mechanism involved loss of nitrogen. Interestingly, these photoelectrodes were also subjected to concentrated light (*ca.* 12 suns). Unfortunately, only reported current–voltage curves were found for that scenario, meaning that no information is available regarding the degradation under those conditions.

From Fig. 4 it is also evident that photoelectrodes without cocatalysts (dashed lines) suffer from rapid photocurrent decay, this is expected as oxidative decomposition could occur due to hole accumulation at the surface. Thus, the lowest photocurrent densities with fastest decay were observed for bare photoelectrodes, as reported for SrTaO_2_N^130^ and TaON,^36^ when compared to their counterparts with deposited catalysts. Currently, there are no available studies on the effect of the different types of necking on degradation. Higashi *et al.* compared the same material (TaON|CoO_*x*_) with and without necking (TaCl_5_),^36^ but due to the insignificant photoactivity observed for the latter case, no conclusion was drawn regarding the effect of necking on photodegradation. It is expected that appropriate necking facilitates electron and hole transport, and consequentially increase the stability of the photoelectrodes.

The two new sets of chronoamperometries included in Fig. 4 show the transient photocurrent of LaTiO_2_N during water photoelectrolysis under 1 and 130 suns at 1.23 V *vs.* RHE. It is worth mentioning that the photocurrent densities did not scale proportionately with the light intensity. This is due to increased ohmic losses *via* the substrate (FTO) and electrolyte, and exacerbated by the increased bubble evolution.^136^ Nevertheless, it can be observed that by increasing the light intensity, the shape of the chronoamperometry seems to shrink in the time domain, suggesting that measurements at higher light intensities could serve as accelerated tests for stability benchmarking in the case of oxynitrides photoanodes. In contrast, when the irradiance is lowered to *ca.* 0.034 suns, the photocurrent of LaTiO_2_N exhibits exceptional stability over time, see Fig. 4 and ref. 134.

**Fig. 5:**
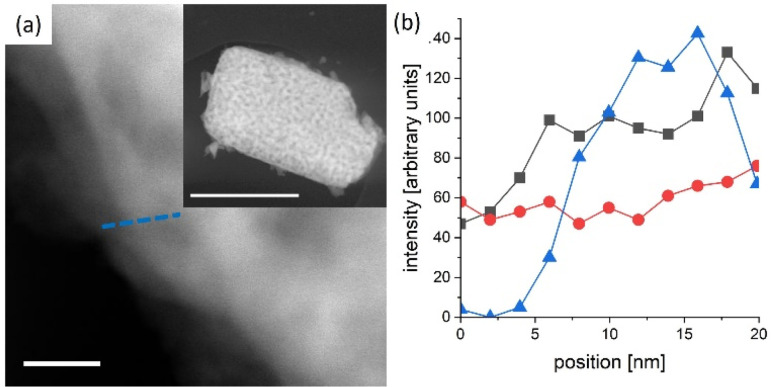
LaTiO_2_N particle scratched from a PEC electrode after 21 h operation. (a) High angle annular dark field scanning TEM image of the edge (scale bar 20 nm) of a particle (see inset scale bar 1 μm) (b) combined scanning TEM EDX/EELS profile along the blue line indicated in (a) triangles in blue denote N K-edge (EELS), black squares and red dots the La L_a_ and Ti K-edge respectively (EDX). The N content is reduced close to the particle edge with respect to the bulk.

In general, reported chronoamperometries indicate that the transient photocurrent follows an exponential decay during the early stages (5 to 20 minutes) continued by a relatively linear decrease over time. Due to the insufficient time lengths of the measurements (1 h) and the lack of standardisation of the operating conditions, it is difficult to establish consistent trends and benchmark the performance in terms of stability. If the degradation mechanism follows an irreversible self-limited oxidative decomposition, the exponential decay seems in agreement with diffusion-controlled oxidation happening in the first few nanometres from the surface of the oxynitride. This assumption is supported by *in situ* measurements performed by Lawley *et al.* for thin film LaTiO_*x*_N_*y*_^113^ and by surface analyses performed by He *et al.* for a similar material (nanotubes of Ta_3_N_5_ obtained by surface anodisation).^54^ According to these two studies, the irreversible oxidative decomposition occurs in the first 3 nm from the surface. He *et al.* listed the reasons why the dominant degradation mechanism is self-limited oxidative decomposition instead of the typically assumed photocorrosion: (i) there are no changes in the diameter of the particles and SEM images showed no sign of change before and after photoelectrolysis, hence, no dissolution seems to be taking place; (ii) there is no build-up of oxides, and the thickness of amorphous structures near the surface (<3 nm) remains constant starting from 6 h until the end of the test (13 h); and, (iii) the XRD patterns, Raman and UV-vis spectra recorded before and after the tests are indistinguishable, meaning that the bulk properties did not change. Our TEM studies on particle based LaTiO_2_N give comparable results (Fig. 5): we observe a reduced nitrogen signal at particle surfaces after 21 h operation by electron energy loss spectroscopy (EELS).

**Fig. 6:**
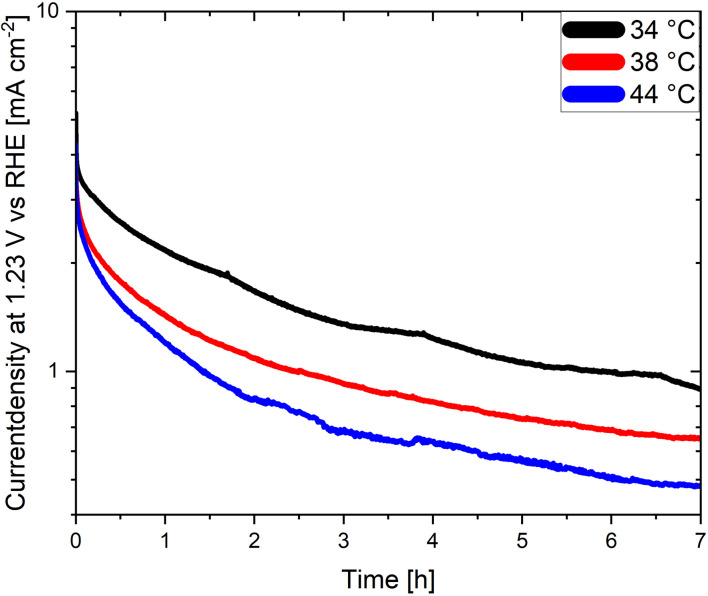
Chronoamperometries of LaTiO_2_N based photoanodes at 1.23 V *vs.* RHE in 0.1 M Na_2_SO_4_ (pH adjusted to 13.4 with NaOH) at various electrolyte temperatures.

An often-neglected parameter in the context of PEC performance and degradation measurements is temperature. Temperature is known to have an important effect on the kinetics and the thermodynamics of the water splitting reaction, as demonstrated in photocatalytic studies where the STH efficiency increases with increasing temperature before reaching a maximum value.^139,140^ Unfortunately, the effect of temperature on the degradation of photoelectrodes has been rarely investigated. Moreover, the electrolyte temperature and/or its evolution during operation is not often indicated even when reporting PEC performance or degradation. This adds additional difficulties for a meaningful comparison of degradation and/or efficiency measurements of different materials considering that the temperature is known to derivate several degree Kelvin from room temperature during long term photoelectrode illumination as a function of the measurement set-up. We present in Fig. 6 long term current density measurements of LaTiO_2_N electrodes at 34 °C, 38 °C and 44 °C demonstrating that the performance of the electrodes changes considerably as a function of these comparatively small temperature variations. Interestingly these preliminary results indicate that the PEC performance decreases with increasing temperature, opposite to what has been reported in photocatalytic studies. Hence, monitoring and reporting the temperature of the electrolyte and, if possible, of the photoelectrode surface should become common practice in future degradation and efficiency-related studies.

In addition to the operating conditions, transport aspects affecting the microenvironment are also important for degradation identification and assessment, especially in particle-based PEC. For example, detailed coupled multi-physical pore-level transport simulations in particle-based LaTiO_2_N photoanodes revealed several orders of magnitude variations in electrolyte concentration and in local current density within a 10–20 μm thick particle-based photoelectrode.^141^ These variations in the local surface conditions are expected to significantly affect the local degradation mechanisms and rates. Similarly, detailed transport modelling within the semiconductor and at the inter-particle interfaces showed^142^ that resistances to the carrier transport resulted in an underutilization of the photoelectrode. Effectively only the photocatalyst particles closest to the substrate contribute to the overall photocurrent, highlighting how inhomogeneity in utilization might result in inhomogeneity in degradation mechanisms and rates.

The studies discussed in this section reveal the degradation challenges faced by oxynitride photoelectrodes for water splitting. While specific details on degradation are not extensively covered in each study, some insights can be drawn. The research collectively suggests that while oxynitrides show promise for solar water splitting, addressing their long-term stability and degradation under operational conditions is crucial for practical applications. It is confirmed that the presence of catalysts has a significant impact on the performance and stability, while the effect of morphology, nature of the substrate, necking procedure, applied electrode potential and crystal orientation still require further research.

## 5 Strategies for stability enhancement

As a first step to stability enhancement, it is important to assess stability consistently and comparably. From the wide range of operating conditions under which the stability of the photoelectrodes is being tested, we propose. as benchmarking method to report long-term chronoamperometries (>6 h) under AM1.5G and 1.23 V *vs.* RHE in 1 M NaOH pH 13.6 (without sacrificial reagents) and accompanied by gas evolution measurements (N_2_, O_2_ and H_2_). It is also advisable to report the retention of photocurrent densities after 1 h, 6 h, and if possible 1000 h as a figure of merit for easier comparison. Because of the fast decay observed in the initial stages of the chronoamperometry, which could stem from capacitive behaviour rather than degradation processes,^143^ it is recommended to calculate photocurrent retentions relative to a point at which the photoelectrode has moved beyond the exponential decay phase. This approach has been suggested previously for the assessment of stability of organic semiconductors.^144^ For this reason, the photocurrent retentions at 60 minutes, as listed in Table 2, were calculated respect to the initial current measured (PR_0_) and after 10 minutes (PR_10 min_) of illumination. As it can be concluded from those values, a comparison of stability using PR_10 min_ could be more useful and reliable than using PR_0_, this is due to the higher sensitivity of the latter metric to experimental conditions, and its susceptibility to effects that are not directly related to degradation processes. However, caution must be taken as the applied electrode potential can have a significant effect on the degradation rate and its mechanism. Unfortunately, little is known of the effect of applied potential on the degradation rates, and only a few thermodynamic predictions have been reported for oxynitrides.^145^ It is also advised to couple the chronoamperometries with other *in operando* measurements taken regularly during the test, with the aim of extracting more information about the stages of degradation. These analyses could include Electrochemical Impedance Spectroscopy (EIS) and Intensity Modulated Photocurrent Spectroscopy (IMPS) as it has been done previously to study the degradation of CdS photoelectrodes;^146^ ICP-MS to account for dissolved species in the electrolyte;^61^ and gas monitoring in order to stablish faradaic efficiencies and the presence, or absence, of desorbed nitrogen in the products.^52,96^

**Fig. 7:**
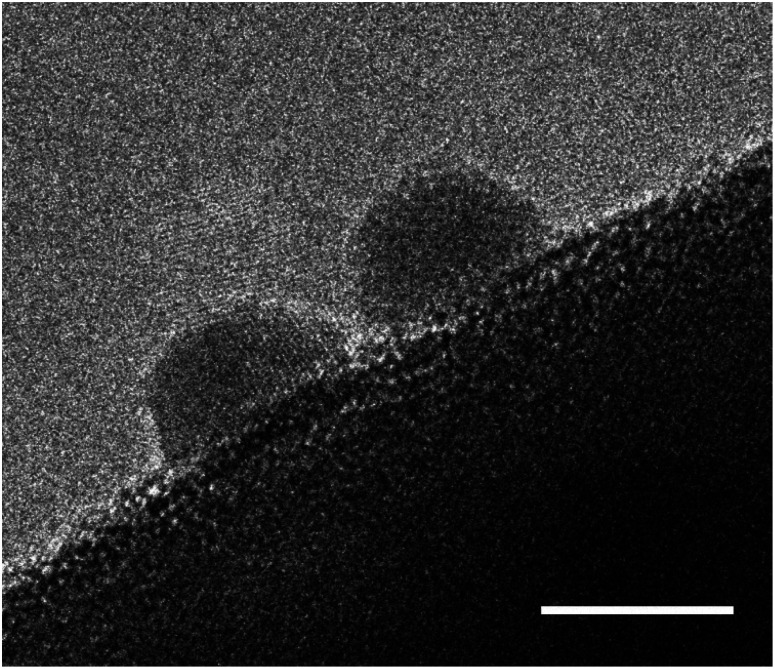
High resolution TEM image of Pt cocatalyst nanoparticles deposited on a BaTaO_2_N photocatalyst particle. The scale bar is set to 5 nm.

Degradation of (oxy)nitride photocatalysts is mainly discussed in the matter of self-oxidation by photogenerated holes. In order to prevent self-oxidation, it is necessary to ensure that photogenerated holes are rapidly extracted and participate in the desired water splitting reaction. Therefore, one of the most effective strategies to mitigate photocorrosion of (oxy)nitrides is the application of cocatalysts. In general, cocatalysts provide active reaction sites, contribute to efficient charge carrier separation, and transfer, and promote the water splitting half reactions, HER or OER.^147–149^ In Fig. 7 cocatalyst particles on an oxynitride particle are shown.

Numerous studies have demonstrated that the application of cocatalysts on (oxy)nitride photocatalysts not only improved the photocatalytic performance but also the stability towards photocorrosion.^36,52,55,132,150^ The dispersion of the cocatalyst has proven to play a crucial role in this context. Loading the cocatalyst after the preparation of the photoelectrode leads to a poor distribution of cocatalyst nanoparticles as it was shown for CoO_*x*_ and IrO_*x*_ TaON photoelectrode.^36^ Similar results were found for the deposition of IrO_*x*_ on LaTaON_2_.^132^ Due to the poor dispersion of the cocatalyst on the photocatalyst, the photocorrosion could not be completely suppressed resulting in a drop in performance over a short period of time. Pre-loading the photocatalyst particles with the cocatalyst resulted in a more homogeneous and fine distribution of nanoparticles which efficiently prevented photocorrosion and improved the stability of the photoelectrode.^36^ Similar to these findings, a study on BaTaO_2_N photoanodes revealed that a combination of pre-loading of CoO_*x*_ on BaTaO_2_N particles and post-loading of RhO_*x*_ resulted in a suppression of photocorrosion.^55^

Cobalt-phosphate (CoPi) is another prominent example of a cocatalyst that promotes the water oxidation reaction and therefore improves the stability of photocatalysts and photoanodes. For instance, bare BaTaO_2_N photoanodes show a rapid decrease in photocurrent in chronoamperometric measurements at 1.23 V *vs.* RHE due to photocorrosion.^151^ However, loading the photoelectrode with CoPi resulted in a stable photocurrent which only decreased by 3% over a measurement duration of 5 h. The reason for this improved stability is that CoPi is deposited as a layer on the photoelectrode which not only causes the improved photocurrent but also acts as protection layer.^151^ Similar improvements have been reported for Ta_3_N_5_ using either CoPi or FeNiO_*x*_ as cocatalysts.^77,103^

The application of protection or passivation layers which separate the photocatalyst from the electrolyte is another strategy to enhance the stability of a photocatalyst (see Fig. 1b).^73,152–154^ The application of hole storage layers, that efficiently harvest and store photogenerated holes, was proposed to prevent the photocorrosion of Ta_3_N_5_ and SrTaO_2_N photoanodes.^32,67,104,155^ To specify, a Ni(OH)_*x*_/MoO_3_ bilayer is reported as a hole storage layer for Ta_3_N_5_ photoanodes which enabled stable water oxidation for 24 h.^155^ Also ferrihydrite has been proposed as a hole storage and protection layer in this context, however, the protection of the photocatalyst towards photocorrosion could be only guaranteed when the photocatalyst was completely covered by the protection layer which — with increasing layer thickness — negatively affected the photocurrent generation due to reduced light absorption by the photocatalyst.^32,104^ With respect to particle-based LaTiO_2_N photoanodes, amorphous Ta_2_O_5_ is applied as a protection layer exhibiting hole conductivity and a high thermal and chemical stability.^73^ In the same study by Landsmann *et al.* a complex post-modification procedure, including TiO_2_ necking, the deposition of the Ta_2_O_5_ protection layer and the application of cocatalysts (NiO_*x*_, CoO_*x*_, and Co(OH)_2_), is suggested to improve the performance and stability of the photoanode, indicating that a complex photoelectrode design is necessary to meet both requirements.

In general, detailed reports by Weng *et al.* and Chen *et al.* are suggested to the reader to give a broader perspective on possible strategies for enhancing the stability of photocatalysts even though they do not focus on (oxy)nitride materials.^48,156^

Factors such as the crystallinity and morphology of the photocatalyst and the reaction environment (electrolyte) are known to affect the performance and stability of a photocatalyst and are therefore relevant to consider when investigating the degradation processes of photocatalysts.^29,48,64^ In addition, some compositional modifications such as substitution or doping are known to increase the performance *via* conductivity improvement,^77,157^ but their influence on compound stability has never been investigated in detail. Moreover, it is necessary to keep in mind that by depositing cocatalysts and protection/passivation layers on the photocatalyst, also potential sites for degradation are created (*e.g.* the chemical or mechanical stability of the cocatalyst particles can be compromised), and light attenuation might occur before photons reach the photoabsorber. Nevertheless, the post-modification of photocatalysts is a promising step towards reaching longevity for photocatalytic and PEC systems.

## 6 Perspective and conclusions

Oxynitride particles are promising photocatalysts for photocatalytic water splitting and as components of particle-based electrodes for photoelectrochemical water splitting. From gas evolution measurements on particles suspended in aqueous electrolytes, it is known that nitrogen evolution takes place in the first hours, which is often, but not in all cases, connected to decreased oxygen or hydrogen evolution rates. Although it has been demonstrated that some degradation is taking place during photocatalytic measurements, it is unclear under which conditions it leads to performance loss. With the aim to unravel the underlying degradation mechanisms, it is necessary to perform more gas evolution rate studies coupled to either *in situ* monitoring of interface and bulk properties, such as composition and structure, or to post-mortem studies. By compared to theoretical predictions of surface activities, a correlation of the measured performance loss with observed structural or compositional material changes might be possible.

Concerning particle-based photoelectrodes the situation is even more complex, since most of chronoamperometric measurements show a seemingly exponential steep drop during the first seconds or minutes, followed by a decrease taking place at a longer time scale that follows a different decay law. Considering the exponential short-term decay, one hypothesis could be that this behaviour is related to a capacitance, for example to the built up of charge at the semiconductor–electrolyte interface^143^ and not directly related to degradation processes. If this is the case, it is important to rethink the way how we determine performance in PEC systems, which is mostly assessed by reporting the photocurrent density at 1.23 V *vs.* RHE under AM 1.5 illumination, usually determined by (cyclo)voltammetry. If the hypothesis is correct, the photocurrent density measured during the first voltammograms contains a significant contribution of capacitive origin, that enhances artificially or masks the actual photoelectrochemical performance of the materials. If this is true, our performance indicators, in terms of photon conversion efficiency and stability, for photoelectrode assessment need to be revised, since we might privilege increased capacitance over photoelectrochemical performance. Based on this scenario, the slower process (>10 min) affecting the current density might be connected to degradation.

Like in photocatalysis, there is some evidence by faradaic efficiency measurements that nitrogen evolution takes place during PEC water splitting. However, it is unclear whether it stops after several hours as observed in photocatalytic experiments, since most faradaic efficiency measurements have been performed for shorter durations (<2 h). To learn more about these processes, it is mandatory to systematically explore the operating parameter space available in PEC systems, such as light intensity or applied bias. By coupling these studies to simulations performed at different time and space scales, there is the potential to identify the physical parameters, such as charge accumulation or local pH changes, that contribute to degradation. Lastly, we believe that it is worthwhile investigating the degradation mechanisms of oxynitrides. Understanding the mechanism behind their degradation can lead to accelerated developments of more stable systems using this promising type of materials, rather than depending on trial-and-error approaches.

## Conflicts of interest

There are no conflicts to declare.
